# Recent advances on the role of monoamine oxidases in cardiac pathophysiology

**DOI:** 10.1007/s00395-023-01012-2

**Published:** 2023-10-04

**Authors:** Nina Kaludercic, Ruth Jepchirchir Arusei, Fabio Di Lisa

**Affiliations:** 1 Department of Biomedical Sciences, University of Padova, Via Ugo Bassi 58/B, 35131 Padua, Italy; 2 Fondazione Istituto di Ricerca Pediatrica Città della Speranza (IRP), 35127 Padua, Italy; 3 Neuroscience Institute, National Research Council of Italy (CNR), 35131 Padua, Italy

**Keywords:** Monoamine oxidases, Mitochondria, ROS, Aldehydes, Cardiac ischemia, Heart failure

## Abstract

Numerous physiological and pathological roles have been attributed to the formation of mitochondrial reactive oxygen species (ROS). However, the individual contribution of different mitochondrial processes independently of bioenergetics remains elusive and clinical treatments unavailable. A notable exception to this complexity is found in the case of monoamine oxidases (MAOs). Unlike other ROS-producing enzymes, especially within mitochondria, MAOs possess a distinct combination of defined molecular structure, substrate specificity, and clinically accessible inhibitors. Another significant aspect of MAO activity is the simultaneous generation of hydrogen peroxide alongside highly reactive aldehydes and ammonia. These three products synergistically impair mitochondrial function at various levels, ultimately jeopardizing cellular metabolic integrity and viability. This pathological condition arises from exacerbated MAO activity, observed in many cardiovascular diseases, thus justifying the exploration of MAO inhibitors as effective cardioprotective strategy. In this context, we not only summarize the deleterious roles of MAOs in cardiac pathologies and the positive effects resulting from genetic or pharmacological MAO inhibition, but also discuss recent findings that expand our understanding on the role of MAO in gene expression and cardiac development.

## Introduction

Among the various enzymes involved in reactive oxygen species (ROS) formation, the following set of features characterize monoamine oxidases (MAOs): (i) specific substrates that are not shared by other ROS producing pathways. For instance, this feature is shared by xanthine oxidase, but not by NADPH oxidases and uncoupled NOS that partake NADPH utilization with many other reactions; (ii) a defined molecular structure; (iii) inhibitors that are currently in use in clinical settings along with genetic manipulations that are not lethal. This set becomes even more unique in mitochondria. Indeed, many mitochondrial pathways generate ROS as a sort of undesired side-product, and most of those pathways, such as respiratory complexes, are deeply involved in bioenergetics. Therefore, they cannot be inhibited pharmacologically or genetically to study ROS formation without altering respiration, ATP synthesis and eventually cell viability. This is obviously not the case with MAOs whose inhibition, far from affecting bioenergetics and viability, can be considered as a reliable way to demonstrate that mitochondria generate ROS in intact cells, tissues or animals. Notably, the utilization of specific substrates allows a direct stimulation of MAOs without interfering with other ROS-producing enzymes. This property, along with a wide array of pharmacological inhibitors and genetic tools, provides a significant advantage characterizing the role of MAOs in pathophysiology as compared to other enzymes generating ROS.

MAO biochemistry and pharmacology have been reviewed extensively [8, 17, 26, 44, 71]. Briefly, the two MAO isoforms, namely, MAO-A and -B, are localized to the outer mitochondrial membrane. Besides structural differences, the two isoforms diverge in specificity towards substrates and inhibitors. MAOs catalyze the oxidative deamination mostly of aromatic amines with relevant roles in neurotransmission and cardiovascular physiology, such as catecholamines and serotonin. Indeed, the major interest in MAO studies has been related to the relevance of their substrates in neurology. The levels of crucial neurotransmitters, such as serotonin and catecholamines are maintained by means of MAO inhibition. This strategy underlies the therapeutic efficacy of MAO inhibitors in several neurological disorders, including depression, Parkinson's and Alzheimer's disease. As discussed below, besides their established role in cell signaling downstream of their receptors, those amines might elicit cytoprotective effects by counteracting inflammation. In addition, the recent description of histone monoaminylation [1] might play a role in gene modulation attributed to MAO.

MAO reaction products are hydrogen peroxide (H_2_O_2_), aldehydes and ammonia. All these molecules have detrimental effects when generated in large amounts due to an increase in MAO activity. Ammonia toxicity is known in the field of encephalopathies resulting from severe liver disease. At the cellular level, ammonia has been described to alter tricarboxylic acid cycle by inhibiting the dihydrolipoyl dehydrogenase component (E3) in pyruvate and α-ketoglutarate dehydrogenases [28]. In addition, ammonia can decrease the formation of succinyl-CoA from α-ketoglutarate by shifting the equilibrium of glutamate dehydrogenase towards glutamate formation. Notably, the inhibition of dihydrolipoyl dehydrogenase in both pyruvate and α-ketoglutarate dehydrogenase is associated with ROS formation [73] that might amplify MAO-induced oxidative stress concomitantly with ammonia generation. In addition to ammonia, H_2_O_2_ can inhibit α-ketoglutarate dehydrogenase indirectly, leading to the reduction in reduced glutathione levels and enzyme glutathionylation [37]. The well-established harmful role of ROS accumulation is paralleled and exacerbated by MAO-generated toxic aromatic aldehydes that are detoxified mostly by aldehyde dehydrogenase 2 (ALDH2) [72]. Therefore, the potential damaging action of an excessive MAO activity is limited by its functional coupling with ALDH2. Indeed, ALDH2 inhibition was shown to both potentiate mitochondrial dysfunction induced by MAO-B [25] and exacerbate cardiac ischemic injury [13]. Notably, since ALDH2 is inhibited by ROS, as shown in nitroglycerine tolerance [56], an excessive MAO activity is likely to contribute to ALDH2 inhibition caused by oxidative stress. Although H_2_O_2_ is a weak inhibitor, ALDH2 is inhibited significantly by 4-hydroxynonenal (4-HNE) and peroxynitrite [38] generated under conditions of oxidative stress contributed by MAO [25]. Consequently, a large accumulation of aldehydes takes place which might disrupt any cell function by irreversible covalent changes of proteins. Thus, an initial large increase in ROS might cause major derangements by boosting aldehyde accumulation. In this respect, it is worth mentioning that, along with semicarbazide-sensitive amino oxidase, MAO-A is involved in glycine, serine and threonine catabolism catalyzing the transformation of aminoacetone into methylglyoxal [57]. However, no information is available on the role of this pathway in cardiac pathophysiology. In this review, we will discuss more recent developments in the field related to MAOs and cardiac disease, as well as focus on the contribution of MAOs to cardiac/cardiomyocyte physiology.

## MAO involvement in cardiac pathology

**Fig.1:**
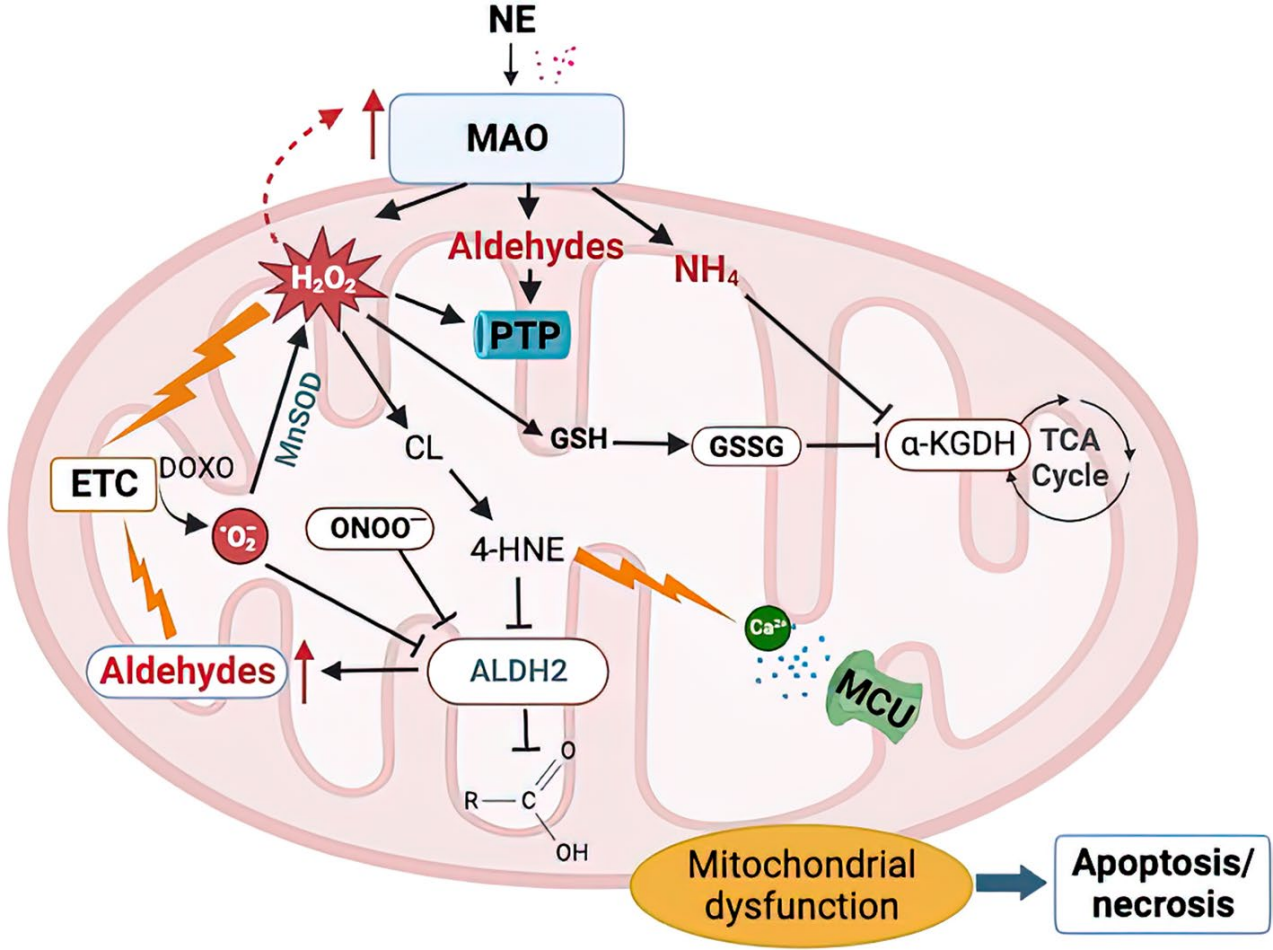
Monoamine oxidases as a source and target of mitochondrial ROS. MAO activity is a major source of mitochondrial ROS and reactive aldehydes that, in turn, induce mitochondrial dysfunction through various pathways. H_2_O_2_, aldehydes, as well as compounds such as doxorubicin, may directly affect the activity of the respiratory chain leading to the generation of superoxide anion which can be converted to H_2_O_2_ by MnSOD. H_2_O_2_ may in turn indirectly affect MAO activity in a feedback loop. These products can also induce PTP opening leading to activation of cell death pathways. MAO-generated ROS are also responsible for peroxidation of mitochondrial cardiolipin leading to the formation of 4-HNE that may target components of the electron transport chain to impair mitochondrial respiration. This, coupled with impaired ALDH2 activity, leads to an accumulation of toxic aldehydes. 4-HNE forms adducts with the MCU resulting in increased channel activity and Ca^2+^ overload. GSH oxidation by H_2_O_2_ can also inhibit other mitochondrial enzymes, such as α-KGDH, through glutathionylation, impairing thereby the activity of the TCA cycle. The figure was created in BioRender. *ALDH2* aldehyde dehydrogenase 2, *CL* cardiolipin, *DOXO* doxorubicin, *ETC* electron transport chain, *4-HNE* 4-hydroxynonenal, *GSH* reduced glutathione, *GSSG* oxidized glutathione, *H*_*2*_*O*_*2*_ hydrogen peroxide, *α-KGDH* α-ketoglutarate dehydrogenase, *MAO* monoamine oxidase, *MCU* mitochondrial Ca^2+^ uniporter, *MnSOD* manganese superoxide dismutase, *NE* norepinephrine, *ONOO*^*−*^ peroxynitrite, *ROS* reactive oxygen species, *PTP* permeability transition pore, *TCA cycle* tricarboxylic acid cycle

Almost two decades ago, the first paper on the role of MAOs in cardiac ischemia/reperfusion injury has been published [7]. Since then, the beneficial effect of MAO inhibition has been further extended and extensively demonstrated in acute and chronic heart diseases, such as ischemia/reperfusion injury, pressure overload-induced heart failure, diabetic cardiomyopathy, or anthracycline-induced cardiotoxicity [4, 10, 11, 18, 25, 27, 40, 60]. The role of MAO-A in ischemia/reperfusion injury and the contribution of their specific targeting by pharmacological and genetic tools to cardioprotection has been extensively reviewed in [24, 26, 33, 34, 51]. More recently, cardiac-specific and tamoxifen-inducible MAO-B knockout mouse has been generated and also shown to present smaller infarct size following ischemia/reperfusion injury [21]. Moreover, the lack of MAO-B in mouse cardiomyocytes reduced the infarct size in male mice, whereas it remained ineffective in female mice [22]. Further studies are necessary to fully understand how cardiomyocyte MAO-B modulates signaling pathways underlying cardioprotection in male vs female mice. One of the primary mechanisms through which MAOs induce pathological changes in the cell is by affecting mitochondria (Fig. 1). Indeed, the direct (i.e., ROS, aldehydes) or indirect (i.e., products of lipid peroxidation) products of MAO activity can directly target and damage mitochondrial components, thereby impairing mitochondrial function. Exacerbated MAO activity in pathological conditions, such as maladaptive hypertrophy or diabetic cardiomyopathy, causes an impairment in the activity of the respiratory chain [25] or directly targets permeability transition pore (PTP) to increase its susceptibility to opening and subsequent activation of the cell death program [18], as shown in isolated adult mouse ventricular cardiomyocytes (AMVMs) or mouse hearts in vivo. Although the immediate and direct consequence of an excessive MAO activity is an accumulation of ROS and aldehydes, recent findings show that mitochondrial Ca^2+^ homeostasis is also altered in a MAO-dependent manner. Indeed, when MAO-A is overexpressed in cardiac myocytes, it results in higher production of H_2_O_2_, peroxidation of cardiolipin, and accumulation of mitochondrial 4-HNE. These abnormalities have been shown to cause an increased uptake of mitochondrial Ca^2+^ due to a MAO-induced modification of the mitochondrial Ca^2+^ uniporter (MCU) complex [47]. The binding of 4-HNE to the MCU leads to the formation of higher order MCU oligomers, thus enhancing Ca^2+^ entry and causing mitochondrial Ca^2+^ overload. This mechanism appears to be particularly relevant in chronic ischemia, as either pharmacological MAO-A inhibition or cardiomyocyte-specific deletion of MAO-A prevented the accumulation of 4-HNE in the mouse heart, the formation of higher order MCU oligomers, and mitochondrial Ca^2+^ overload [47]. Therefore, an initial increase in MAO-induced oxidative stress under pathological conditions is likely to be followed by mitochondrial Ca^2+^ overload and PTP opening paving the way to a loss of cardiomyocyte function and viability.

In addition to modulating mitochondrial Ca^2+^ levels, MAO activity is associated with aberrant intracellular Ca^2+^ cycling also in other pathological settings, such as doxorubicin-induced cardiotoxicity in mice [4]. In this context, MAO inhibition prevented mitochondrial and cardiomyocyte dysfunction. Moreover, pargyline prevented doxorubicin-induced derangements in excitation–contraction coupling by preserving both cardiomyocyte structure and intracellular Ca^2+^ homeostasis, suggesting that MAO-dependent ROS are likely to target intracellular Ca^2+^ stores. Indeed, previous findings showed that a primary increase in mitochondrial ROS using mitochondria-targeted paraquat has a strong impact on the intracellular Ca^2+^ homeostasis and consequently cardiomyocyte function, using neonatal rat ventricular cardiomyocytes (NRVMs) [5]. Taken together, these results suggest mitochondrial ROS act upstream of the impairments in the cytosolic Ca^2+^ homeostasis, adding onto the well-characterized interplay between ROS and intracellular Ca^2+^ homeostasis in cardiac physiology and disease [12]. Whether MAO-generated ROS directly target intracellular Ca^2+^ channels or Ca^2+^ handling proteins, or if they impact signaling pathways involved in the regulation of Ca^2+^ levels, remains to be elucidated.

### MAO and regulation of gene expression

MAO-induced ROS formation has been linked to the dysregulation of miRNA expression. Gene and miRNA expression profiling in cardiac tissue from streptozotocin-treated mice, administered with either vehicle or MAO inhibitor pargyline for 12 weeks, revealed that MAO activity in type I diabetic mouse hearts increased miR-133a-3p, -193a-3p, and -27a-3p expression, among others [10]. Bioinformatic analyses identified insulin-like growth factor receptor 1 (*Igf1r*) and several components of the signaling pathway downstream of this receptor as targets for these miRNAs. Specifically, using HL-1 cells and NRVMs, miR-27a-3p was shown to directly target inositol polyphosphate 4 phosphatase type 1A (*Inpp4a*), whereas miR-193a-3p regulated the levels of growth factor receptor-bound protein 10 (*Grb10*). Of note, these two proteins are involved in the regulation of the PI3K/AKT pathway. Indeed, MAO-dependent regulation of these miRNAs in diabetes had a major effect on the activation of the IGF1R/PI3K/AKT axis in the diabetic mouse heart [10], revealing an additional layer of complexity in the modulation of pro-survival pathway activation. The mechanisms governing these MAO-dependent alterations in miRNA levels are less clear. ROS can directly oxidize RNA molecules, including primary miRNA transcripts (pri-miRNAs) and pre-miRNAs [52, 58, 70], thereby inducing oxidative modifications to RNA molecules that may influence their processing and maturation. On the other hand, it cannot be excluded that ROS generated by MAO might modulate the activity of transcription factors involved in miRNA biogenesis and processing, or that MAO-generated ROS can influence RNA-binding proteins, thereby affecting the stability and turnover of miRNAs [29, 53]. It is important to note that the specific impact of MAO activity on miRNA expression may vary depending on the cell type, tissue, and context. The interplay between MAO, ROS, and miRNA expression is a complex and dynamic process that requires further investigation to fully understand its implications in various physiological and pathological conditions.

### MAO and regulation of autophagy

Transcriptomic analysis of diabetic mouse hearts also showed that the mTOR signaling pathway was highly affected by MAO-generated ROS [10]. ROS may act as the principal intracellular signal transducers contributing to the induction of autophagy via direct oxidation of molecules within the autophagy machinery, such as Atg4 [50], or by affecting other players in the complex autophagy machinery. Indeed, several studies suggested that MAO-A-induced oxidative damage to mitochondria is amplified by impaired cellular quality control mechanisms [31, 49, 62]. Exacerbated MAO-A activity has been shown to target transcription factor-EB (TFEB)-mediated lysosomal biogenesis in transgenic MAO-A overexpressing mouse hearts or NRVMs, thereby impairing lysosomal function [49]. In addition, MAO-generated ROS also lead to p53 activation and consequent mitochondrial oxidative damage due to the inhibition of parkin-mediated mitophagy by p53 [31]. While elevated MAO activity has been associated with impaired autophagy, potentially leading to cellular dysfunction and contributing to various pathological conditions, in some cases, increased MAO-A activity has been shown to promote protective autophagy through Bcl-2 phosphorylation and associated increase in Beclin 1 levels, to mediate the removal of damaged macromolecules/organelles [59]. The latter study has been performed in human SH-SY5Y neuroblastoma cells; thus, it remains to be elucidated whether the same holds true in cardiac myocytes. Overall, the interplay between MAO activity and autophagy represents a complex and intriguing area of research, with implications for understanding cellular homeostasis and the development of novel therapeutic approaches for diseases involving autophagy dysregulation. Further investigations into the molecular mechanisms underlying the relationship between MAOs and autophagy may provide valuable insights into cellular health and disease.

### MAO involvement in ageing

Age-associated frailty is worsened by cardiac ageing characterized by an increase in sympathetic tone along with a decrease in left ventricular diastolic pressure and an increased occurrence of arrhythmias and fibrosis. At the cellular level, senescence refers to a permanent cell cycle arrest that is characterized by various abnormalities, including an impaired mitochondrial function and biogenesis [16, 48]. Since oxidative stress is involved in age-related diseases and cellular senescence, MAO has gained interest as a potential player in cardiac aging. An initial support was provided by showing that MAO-A expression is increased in the rat heart in an age-dependent manner [32]. Then, cardiomyocyte-specific MAO-A overexpression in mouse hearts was demonstrated to result in cardiac derangements mimicking those associated with ageing. First, increase in H_2_O_2_ levels in cardiomyocytes overexpressing MAO-A was concomitant with p53 accumulation, mitochondrial dysfunction and cell death. Those alterations were prevented by both MAO-A inhibitor clorgyline and N-acetylcysteine (NAC) demonstrating the causal relationship with ROS generation and MAO activity [62]. In a later report, oxidative stress induced by MAO-A overexpression in NRVMs was shown to cause DNA damage leading to the activation of the DNA-damage response (DDR), as demonstrated by the increase in the levels of its downstream effectors and the activity of senescence associated-β galactosidase [31]. Interestingly, the authors found that MAO‐A, but not MAO‐B, was upregulated in AMVMs isolated from 20‐month‐old compared to 3‐month‐old cardiomyocytes. Further support to MAO-A involvement in cardiac ageing was provided by demonstrating that cardiomyocyte-specific MAO-A overexpression in mouse hearts induces telomere damage and hypertrophy. This phenotype was prevented by NAC suggesting that it was ROS-dependent. Furthermore, these cells also secreted senescence-associated secretory phenotype (SASP)-like signaling molecules that induced senescence in other cell types [3]. MAO-A overexpression was also shown to recapitulate mitochondrial derangements associated with senescence. Besides mitochondrial dysfunction, MAO-overexpression in cardiomyocytes resulted in a downregulation of PGC-1α, coactivator of peroxisome proliferator-activated receptor-γ and a master regulator of mitochondrial biogenesis [62]. Taken together, these studies validate the importance of MAO–ROS in cardiac ageing.

### MAOs and inflammation

The increase in ROS and aldehydes, and the decrease in catecholamines support an exacerbating role of MAO in inflammation, a common denominator of most age-related diseases. Consequently, potential anti-inflammatory actions of MAO inhibitors have been reported [39], justified by both a decrease in toxic end-products and an increase in availability of substrates, especially catecholamines. On one hand, all three products of MAO activity, namely, H_2_O_2_, aldehydes and ammonia, have been described as inflammatory drivers that, when in excess, along with mitochondrial dysfunction play relevant roles in chronic and autoimmune diseases [42]. On the other hand, increased catecholamines due to MAO inhibition have been shown to reduce lymphocyte proliferation in a process mostly dependent on β_2_ adrenergic receptors (ARs) [43]. However, it is worth pointing out that the anti-inflammatory roles of catecholamines due to MAO inhibition have been described in joint diseases but not yet in cardiovascular pathologies. Another aspect relating MAO with inflammation is the induction of both MAO-A and MAO-B expression by glucocorticoids [43] that might underlie undesired effects of anti-inflammatory treatments based on corticosteroids.

### MAO involvement in other cells types relevant for the heart

It is worth mentioning that, although MAOs play an important role in cardiac myocytes, the most mitochondria-rich cells in the heart, other cell types are affected as well. For instance, MAO-dependent ROS formation is a key mediator of endothelial dysfunction in experimental models mimicking hypertension or type I diabetes [54, 55]. In the context of hypertension, histamine was shown to trigger MAO-dependent impairment in vasorelaxation, although the link between histamine and MAO activity was not known at the time [55]. Soon after, it became clear that N^1^-methylhistamine is a substrate for MAO and can thus fuel MAO activity when histamine is available [15]. Other studies have highlighted the role of MAO-A in regulating vascular function by affecting vascular smooth muscle cells [14, 41]. MAO activity also appears to be relevant in immune cells. For instance, MAO inhibition prevented mast cell degranulation and activation of pro-fibrotic signaling, ultimately resulting in reduced fibrosis in diabetic hearts [18]. Although MAO activity did not affect NLRP3 inflammasome activation and IL-1β formation in cardiomyocytes exposed to the diabetic milieu [18], MAO-B activity was shown to be crucial for NF-κB and NLRP3 inflammasome activation in bone-marrow-derived macrophages [46]. This finding is of importance, considering that macrophages may infiltrate cardiac tissue in several pathological conditions affecting the heart [23, 35].

## MAO involvement in cardiac physiology

**Fig. 2:**
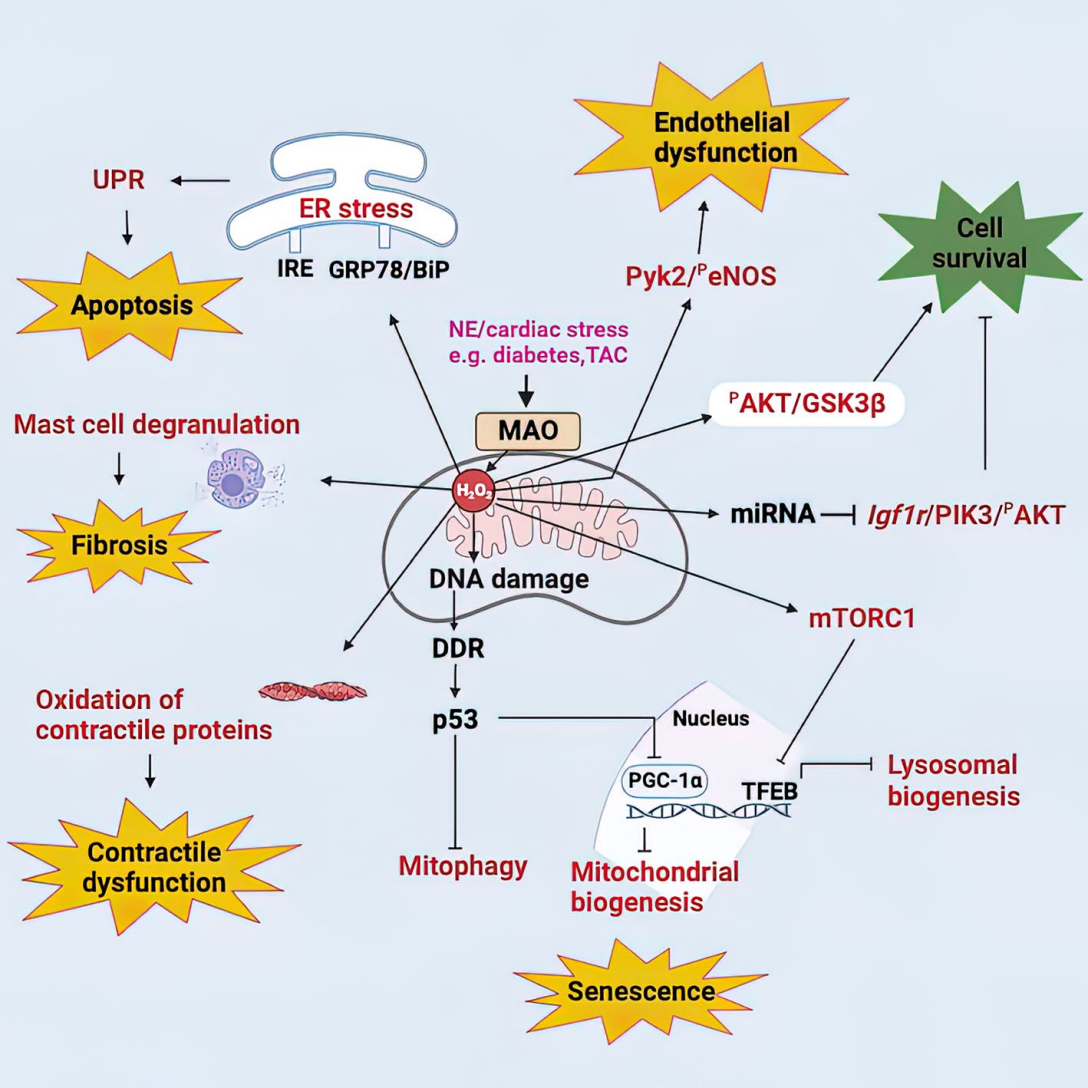
The role of MAO in cardiac pathology and physiology. MAO is a source of ROS and aldehydes, associated with various cardiac pathologies. MAO-generated ROS induce ER stress, consequently leading to the activation of UPR. When prolonged, this leads to the activation of apoptotic pathways. MAO-dependent ROS can also induce contraction/relaxation dysfunction, either through direct oxidation of myofibrillar proteins or by inducing mast cell degranulation and consequent fibrosis. ROS have also been shown to activate Pyk2 which in turn phosphorylates eNOS rendering it inactive and thereby affecting endothelial function. Furthermore, MAOs have been implicated in the upregulation of miRNAs which post-transcriptionally regulate the expression of *Igf1r* and other downstream proteins leading to the downregulation of IGF1R/AKT signalling pathway, consequently affecting cell survival. MAO-dependent ROS have also been associated with the senescent phenotype through accumulation of cytosolic p53. This arrests parkin-mediated mitophagy, while reduced expression of PGC-1α, a co-activator of PPAR-*γ*, impairs mitochondrial biogenesis in a MAO-dependent manner. Activation of mTORC1 by ROS negatively regulates lysosomal turnover due to the inhibition of TFEB translocation into the nucleus. On the flip side, MAO activity also has a physiological role in the heart. It is a source of ROS essential for correct cardiomyocyte differentiation through the activation of the AKT/GSK3β pathway. The figure was created in BioRender. *AKT* protein kinase B, *DDR* DNA damage response, *eNOS* endothelial nitric oxide synthase, *ER* endoplasmic reticulum, *GRP78/BiP* glucose-regulated protein GRP78/immunoglobulin heavy chain-binding protein, *GSK3β* glycogen synthase kinase-3β, *Igf1r* insulin-like growth factor 1 receptor, *IRE* inositol-requiring enzyme 1 α, *MAO* monoamine oxidase, *mTORC1* mammalian target of rapamycin complex 1, *PGC-1α* proliferator-activated receptor-gamma coactivator 1α, *PPAR-γ* peroxisome proliferator activated receptor, *Pyk2* proline-rich tyrosine kinase 2, *ROS* reactive oxygen species, *UPR* unfolded protein response

MAO activity plays a major role in the intracellular signaling network, thus regulating cardiomyocyte homeostasis (Fig. 2). Recently, intracellular ARs have been identified and shown to influence cardiomyocyte response to catecholamines and signaling [9, 36, 64, 67–69]. Importantly, there is a functional pool of β_1_ARs residing on the sarcoplasmic reticulum (SR-β_1_ARs), which associate with SR Ca^2+^ ATPase 2a and whose stimulation promotes local protein kinase A (PKA) activity for phosphorylation of phospholamban and enhances Ca^2+^ cycling in excitation–contraction coupling [64]. Given the structural vicinity of SR and mitochondria and considering the localization of MAO-A at the mitochondrial outer membrane, MAO-A is the likely candidate for regulating intracellular catecholamine levels and fine-tuning the SR-β_1_AR signaling during the fight-or-flight response. In a very elegant study, Wang and colleagues showed how organic cation transporter 3, responsible for catecholamine uptake into the cardiac myocyte, and MAO-A dynamically control intracellular β_1_AR signaling and cardiac excitation–contraction coupling under catecholamine stimulation [66]. Both cardiomyocyte-specific MAO-A deletion and pharmacological inhibition in mice selectively enhanced the local β_1_AR–PKA activity at the SR but not at the plasma membrane, and augmented phosphorylation of phospholamban, Ca^2+^ cycling, and myocyte contractile response. Overexpression of MAO-A suppressed the SR–β_1_AR–PKA activity and PKA phosphorylation, highlighting the importance of this regulatory mechanism in heart failure, a condition in which intracellular β_1_AR signaling is desensitized [65]. In addition, this regulatory mechanism is likely in place to prevent excessive stimulation of cardiac βARs by catecholamines, thereby avoiding the occurrence of arrhythmias.

Recently, our research group discovered a specific context in which the formation of ROS dependent on MAO is crucial for triggering signaling pathways and ensuring the correct differentiation of cardiomyocytes [19]. To investigate the hypothesis that ROS generated by MAOs play a direct role in regulating human cardiac specification, we conducted experiments to observe whether MAO-A contributes to mitochondrial ROS formation during the differentiation of human induced pluripotent stem cells (hiPSCs) into cardiomyocytes (hiPSC-CMs). Of note, MAO-A appears to be the predominant isoform expressed in the hiPSCs, whereas MAO-B is absent and appears only after 40 days of differentiation, in mature hiPSC-CMs [19]. Our findings revealed that hiPSC-CMs with MAO-A knockout or knockdown exhibited impaired sarcomere structure and function, along with reduced levels of mitochondrial ROS. This reduced ROS formation due to the lack of MAO-A led to a decrease in the phosphorylation of AKT/GSK3β, reduced WNT expression, and hindered the downstream activation of cardiac transcription factors MESP1 and NKX2.5. We further confirmed this cause-and-effect relationship by demonstrating that either adding exogenous H_2_O_2_ to MAO-A knockout cells or reintroducing MAO-A enzyme improved AKT/GSK3β phosphorylation, increased NKX2.5 and WNT3A transcript abundance, and restored the proper cardiomyocyte structure and Ca^2+^ homeostasis. While the involvement of MAOs in differentiation processes and development had been mainly studied in the brain, where MAO-A inhibition affects neurogenesis due to elevated serotonin levels [63], our study by Di Sante et al. presents evidence that MAO-A-driven ROS generation is vital for activating the AKT and WNT signaling pathways during the early stages of human cardiomyogenesis and for producing fully functional cardiomyocytes [19]. These findings suggest that, as with many other ROS sources, MAO-dependent ROS formation is beneficial up to a certain threshold and identify a dual, hormetic role for ROS produced by MAO-A.

## Evidence supporting the role of MAO in cardiovascular disease (CVD) in patients and its potential as a novel therapeutic target for the treatment of CVD

To date, only a limited number of studies have investigated the involvement of MAO in patients with CVD. Anderson et al. conducted a study revealing that MAO plays a significant role as a source of ROS in human atrial myocardium, generating ROS levels approximately 10 times higher than those produced by the mitochondrial respiratory chain [2]. Notably, the Authors demonstrated that MAO activity is a crucial factor influencing myocardial redox balance in patients and represents a major risk factor and predictor for postoperative atrial fibrillation [2]. MAO-dependent serotonin degradation was increased in patients with aortic valve stenosis, together with platelet activation and arterial circulating serotonin, suggesting that it may have a role in the pathogenesis of aortic valve stenosis, as well as valve fibrosis and adverse ventricular remodeling [45]. Furthermore, Manni et al. measured MAO activity in the left and right ventricles of human hearts, including non-failing (NF) and end-stage ischemic (IHD) and non-ischemic failing hearts [30]. In ventricles affected by IHD, there was a notable rise in both MAO isoforms (MAO-A/B) in terms of activity and expression levels. In addition, actin oxidation was significantly higher in both failing ventricles, and this increase was closely linked to the activity of MAO-A and correlated with changes in cardiac functional parameters. Another recent study showed that gene expression of both MAO-A and -B is more than doubled in cardiomyocytes differentiated from human induced pluripotent cells (hiPSC-CMs) from a Duchenne muscular dystrophy (DMD) patient, affected by cardiomyopathy, vs healthy controls [6]. The DMD hiPSC-CMs show a cardiomyopathy-related transcription profile, that could be corrected using a CRISPR/Cas9 gene editing approach to rescue dystrophin expression. Of note, the corrected DMD hiPSC-CMs show normalized levels of both MAO-A and -B, along with other genes. Taken together, these findings strongly suggest a close connection between MAO-A-dependent ROS generation and cardiomyocyte dysfunction, highlighting MAOs as a promising new therapeutic target for heart failure.

## Conclusive remarks

MAOs play an important role in cardiac pathophysiology, as demonstrated by studies employing animal experimental models as well as human samples. MAO-mediated effects in the heart include the regulation of catecholamines and biogenic amines levels, as well as the impact of products resulting from MAO activity on cardiomyocyte structure and function. Numerous studies have highlighted the deleterious effect of MAO-dependent ROS and aldehyde formation on mitochondrial and cardiac function. These findings strongly suggest that MAOs may represent a promising therapeutic target. However, MAO inhibitors have a negative reputation due to off-target effects, such as hypertensive crises, better known as the “cheese reaction.” This reaction can occur in patients treated with irreversible MAO-A inhibitors after consuming foods rich in tyramine, such as cheese, red wine, fava beans, soy sauce, or chocolate [71]. It is worth mentioning that these side effects are associated with the now outdated irreversible MAO-A inhibitors that are no longer used in clinical practice. Currently, molecules such as moclobemide (a reversible MAO-A inhibitor) or safinamide (a reversible MAO-B inhibitor) are employed in clinical settings and are devoid of the aforementioned side effects [20, 61]. Therefore, reconsidering MAO inhibitors as a potential tool for treating CVD might be worthwhile.

## Data Availability

There is no data associated with this manuscript.
